# Distance Field-Based Convolutional Neural Network for Edge Detection

**DOI:** 10.1155/2022/1712258

**Published:** 2022-03-03

**Authors:** Dadan Hu, Hongbo Yang, Xia Hou

**Affiliations:** ^1^School of Automation, Beijing Information Science and Technology University, Beijing 100192, China; ^2^Computer School, Beijing Information Science and Technology University, Beijing 100192, China

## Abstract

In this paper, we first propose an accurate edge detector using a distance field-based convolutional neural network (DF-CNN). In recent years, CNNs have been proved to be effective in image processing and computer vision. As edge detection is a fundamental problem among them, we try to improve the accuracy of edge detection based on the deep learning framework. The proposed network combines a feature extraction backbone that can fully exploit the multiscale and multilevel information of the edge with the supervised training of the distance field branch to realize the accurate end-to-end object edge detection. The distance field branch is applied to predict the Euclidean distance from nonedge points to the nearest edge point in the feature maps. And the distance information embedded in the predicted distance field map can effectively improve the accuracy of edge detection. The network is trained to minimize the weighted sum of the distance field branch loss and the cross-entropy loss. Our experimental results show that the proposed edge detector achieves better performance than previous approaches and the effectiveness of the proposed distance field branch.

## 1. Introduction

Edge detection is one of the low-level challenging tasks in the field of computer vision. The improvement of the edge detection technology can promote the development of medium and high-level visual tasks (e.g., object detection [1, 2] and image segmentation [3, 4]). Generally, a good edge detection algorithm has the following characteristics: (i) effectivity, it can detect the edge and is effective for various problems; (ii) integrity, the closed and continuous contour of the interested object region can be obtained, which has no breakpoints and discrete points; (iii) accuracy, the obtained edge is as close as possible to the true edge.

A large amount of background and structural information contained in the image is important for traditional edge detection methods which usually give priority to the underlying features such as color, brightness, and gradient. The traditional edge detection methods can be summarized as follows: (i) The early pioneering methods, such as PB [5], Sobel operator [6], and the widely used Canny operator [7]; (ii) Konishi et al. [8] expressed edge detection as a statistical inference based on data-driven technology and realized edge detection by using the joint probability distribution of image features. Martin et al. [5] input the brightness, illumination, texture, and other local features of the image into the logistic regression classifier for edge judgment. The performance of the method that designed features manually based on information theory has been greatly improved compared with the early pioneering methods, but its cost is high, the steps are tedious, and the real-time performance is not good; (iii) Structured edge detection algorithms that contain the SE (structured forest edge detection) algorithm [9], etc. The limitations of the traditional edge detection algorithms are inefficiency and low accuracy so that they cannot be widely applied.

In recent years, with the development of computing power, CNNs have great advantages in automatic learning of natural images so that it becomes increasingly popular in a variety of computer vision tasks, such as image classification [10–12], semantic segmentation [13, 14], and instance segmentation [15]. More and more researchers begin to use CNNs to detect the edge of objects, in which some successful algorithms have sprung up. In 2015, Shen et al. [16] introduced a deep convolutional neural network (DCNN) to detect the edge and proposed an edge detection algorithm named deep contour. Bertasius et al. [17] proposed an end-to-end network architecture deep edge, which combines the local and global information of images to significantly improve the accuracy of edge detection. Xie and Tu [18] studied HED that solved two important problems: (i) the training and prediction based on the whole image; (ii) multiscale feature learning. Previously, methods based on CNN usually only adopted the feature information of the last layer of each convolution stage. In 2017, Liu et al. [19] proposed RCF to combine features from each CNN layer efficiently. Recently, Su et al. [20] considered that the edge detection algorithms based on CNN can achieve high performance because it depends on the large pretrained CNN backbone; however, it will consume a lot of memory and energy. In addition, a simple, lightweight, and effective architecture named pixel difference network (PiDiNet) for efficient edge detection was proposed. Although the processing speed of PiDiNet is fast, the accuracy of edge detection is not high enough.

Based on the observations of the well-known edge detection algorithms (e.g., HED [18], RCF [19], and BDCN [21]) which adopt pixelwise binary cross-entropy loss, we consider that these methods ignore a factor that is the distance between background points and edge. Concretely speaking, in the loss of the aforementioned model, the errors on all points in the image provide the same contribution to network tuning. Actually, the distance information of each pixel to the edge is vital. Further, the distance information is applied to the network by introducing a distance field branch to enhance the accuracy of edge detection.

## 2. Related Work

Since the problem of edge detection has been regarded as one of the most fundamental problems in computer vision, researchers have devoted themselves to it for nearly 60 years, and they have emerged a large number of approaches. Generally speaking, these methods were roughly divided into two categories: classical traditional methods and deep learning-based methods. Here are some of the most representative approaches in the past few decades.

The classical traditional edge detection algorithms often focus on the color, gradient, and texture underlying features of images. Robert operator [22] is an operator that uses a local difference operator to find edges. Because the Robert operator usually generates a wide response in the region near the edge of the image, the accuracy of edge detection is not very high. Sobel operator is a form of a filtering operator, which is used to extract edges. It can use a fast convolution function, which is simple and effective. But the Sobel operator does not strictly distinguish the foreground of the image from the background; in other words, the Sobel operator is not based on the image gray processing because the Sobel operator does not strictly simulate human visual physiological characteristics, so the extracted image edge is sometimes not satisfactory. The Canny operator is a multistage optimization operator with filtering, enhancement, and detection, and has strong robustness. Before processing, the Canny operator first uses a Gaussian smoothing filter to smooth the image and remove the noise. The Canny segmentation algorithm uses the finite difference of the first partial derivative to calculate the gradient amplitude and direction. However, the poor accuracy of these methods makes it difficult to be adopted in today's applications.

With the rapid development of deep learning in recent years, a series of deep learning methods have been proposed for edge detection, in which the RCF based on HED is one of the best edge detection algorithms. Here, we briefly review the structure of the HED and RCF.

The highlights of HED are briefly summarized as follows: (i) image to image. The algorithm learns automatically throughout the whole process without any other operation, and when we input an image into the model, we can get the result directly; (ii) based on the improvement of FCN and VGG, six losses were simultaneously extracted for optimization training, edges of different scales were output through multiple side outputs, and then the final edge output was obtained through a trained weight fusion function. We note that in paper HED, the six losses are trained simultaneously. In the prediction stage, the output result of the last layer can be directly taken as the final result. We can also take the output of all layers and average it for the final result, and the advantage of this is that it will further improve the accuracy, but the disadvantage is that it will increase the time when additional operations are added; (iii) in the training process, edge detection is actually a binary classification task for each pixel. Most of the pixels are nonedges and only a few are edges. In order to balance the positive and negative samples, the authors introduce the class-balanced cross entropy. It is obvious that the six losses that are simultaneously trained are complicated, and the class-balanced cross-entropy strategy assumes that each pixel contributes equally to the loss so that there is room for improvement in the edge detection task.

**Figure 1:**
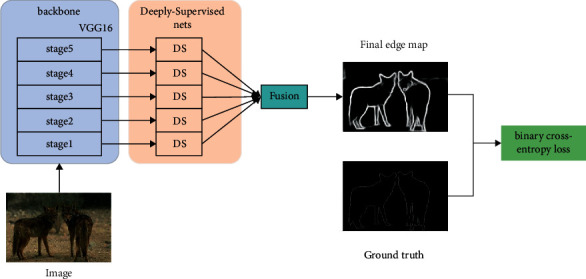
Structure of RCF.

As shown in Figure 1, the RCF is made up of a backbone that adopts all the convolutional layers of VGG16, deeply supervised nets, and fusion modules. The backbone is divided into five stages; with this fully convolutional structure, it can extract edge features automatically. The deeply supervised nets of RCF conduct supervised learning for each stage and output a predicted edge map for each stage so that the model can converge better and faster. The fusion module of RCF fuses the five edge maps which are output by deeply supervised nets with a 1 × 1 convolution layer. Because the final edge map of RCF contains the feature information of each layer of the backbone, it is better than the edge map of HED that only use some feature information. The same problem with HED is not taken into account that each pixel has a different degree of influence on the loss.

The distance field introduced in this paper is essentially a function that obtains the distance between the nonedge points and the nearest edge point. It can be expressed as

(1) $$\begin{array}{c} D\left(i,j\right)=\left\{\begin{array}{cc} 0, & \text{if value}\left(i,j\right)=0,\\ \text{min}\left(\sqrt{{\left(i-m\right)}^{2}+{\left(j-n\right)}^{2}}\right), & \text{if value}\left(i,j\right)\neq 0, \end{array}\right) \end{array}$$

in which *D*(*i*, *j*) and value(*i*, *j*) denote the value of the pixel (*i*, *j*) in the distance field map and the value of the pixel (*i*, *j*) in the edge map respectively. The horizontal and vertical values of edge points in the image were represented by *m* and *n*.

**Figure 2:**
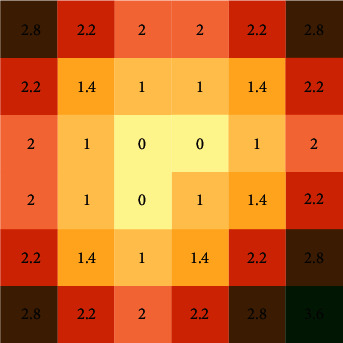
A distance field example.

A distance field example is illustrated in Figure 2, in which we produce a distance field by calculating the Euclidean distance between a given pixel and the other pixels. In the traditional convolutional neural network, the task of edge detection is a binary classification for each point in the image. However, sometimes, the results are not satisfactory. To improve the accuracy of edge detection, the edge map in the distance field map is embedded, and we will obtain rich distance information. When misjudgment is made at the points farther from the edge, the loss function is so large that it can converge quickly. The next section describes our network in detail.

## 3. Distance Field-Based Convolutional Neural Network (DF-CNN)

**Figure 3:**
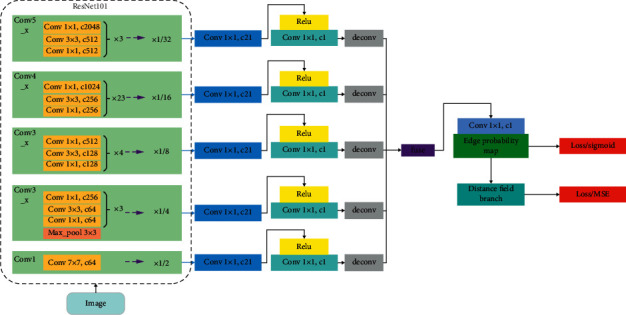
Our DF-CNN network architecture. The input is an image with arbitrary sizes, and the model outputs a predicted edge probability map and a predicted distance field map in the same size, respectively.

**Figure 4:**
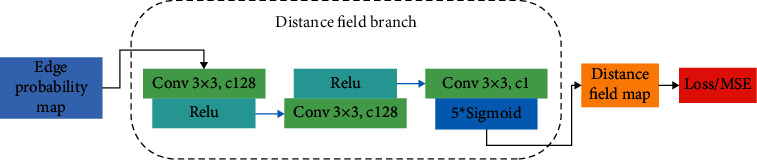
Detail of the distance field branch. The edge probability map generated from REFN is fed into the distance field branch to obtain a predicted distance field map.

In this section, we introduced our proposed distance field-based convolutional neural network, termed DF-CNN, which receives an image as input and then generates the edge probability map and the predicted distance field map as output. DF-CNN can be split into two subnetworks (as shown in Figures 3 and 4): ResnNet101 feature extraction network (RFEN) and distance field (DF) branch. While RFEN is fed with the source image, DF is fed with the edge probability map which is the output of RFEN.

### 3.1. Network Architecture

After investigating many pieces of literature in the field of deep learning, the design idea of this model is derived from the RCF network, and the module of RCF is improved accordingly. As is shown in Figure 3, we choose ResNet101 as the backbone of image feature extraction.

Even though DF-CNN is inspired by RCF, major differences are described as follows:

- We use ResNet101 instead of VGG16 [11] and cut off all the fully connected layers. We can see that the ResNet achieves state-of-the-art performance in the ImageNet large-scale visual recognition challenge of 2015; with sufficient datasets in this work, it can be believed that ResNet101 can further improve performance. To be honest, due to the rapid development of classification in ImageNet, there are currently better backbones than ResNet; of course, many tricks such as attention mechanism can also improve the performance; but in this paper, what we are concerned about is the effect of the distance field branch. In addition, we do not do too much research on the backbone network.
- Each 1 × 1-21 Conv layer is connected to the ReLu layer. Then, a deconv layer is connected to the upsample corresponding feature map so that the model can generate an edge probability map that is the same size as the original image.
- For simplicity, a cross-entropy loss/sigmoid layer is only connected to the fusion layer in the network. We do not care about the learning results of each intermediate feature layer but only pay attention to the learning of the fusion layer.
- The loss function of the distance field branch is added to the cross-entropy loss function of the predicted edge probability map and ground truth (true edge probability map) as the loss function of the whole network. In this way, the distance factor from nonedge points to edge can be contributed to the model loss.

We combine the distance field branch into a holistic end-to-end framework, in which the distance field branch monitors the parameter learning of the entire network. ResNet101 backbone obtains the feature information of each convolution layer; although after fusion, the fusion layer will have rich edge information; still, there will be some deviation; here, to join a branch, by monitoring the distance from the non-edge points to the edge points of the predicted edge probability map, the whole network is made to further study, and more accurate edge map is obtained.

### 3.2. Distance Field Branch

DF-CNN has been proposed to generate a predicted edge probability map. To improve the accuracy of edge detection, a key component of DF-CNN is the distance field branch, as appreciated in Figure 3, the output from the RFEN block feeds the DF; of course, we will process the output of RFEN, the threshold value is set as 0.25, and the probability value of the edge map is less than or equal to the threshold value; 1 is set, if not, 0 is set. The DF consists of some basic CNN layers. The DF is set as shown in Figure 4. Kernel size and channel of the conv1 layer is 3 × 3 and 128, respectively, followed by a ReLU activation function; kernel size and channel of the conv2 layer is 3 × 3 and 128, respectively, followed by a ReLU activation function; kernel size and channel of the conv3 layer is 3 × 3 and 1, respectively, followed by a sigmoid activation function; the last layer gives a predicted distance field map with the same size as the true distance field map.

**Figure 5:**
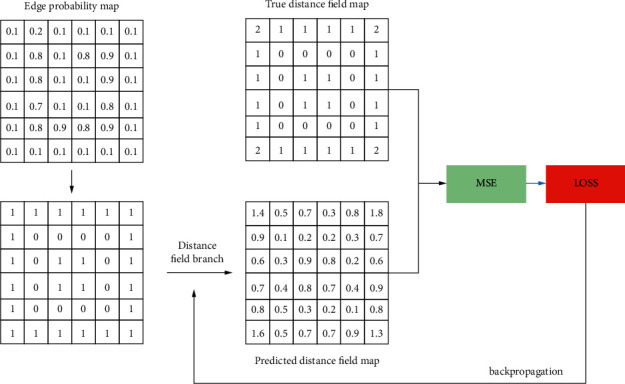
Distance field branching example demonstration.

If we go through DF, we get the predicted distance field map that corresponds to the predicted edge probability map. As appreciated in Figure 5, we show a working diagram of a distance field branch. For the predicted edge probability map obtained from RFEN, we set the probability threshold value as 0.25. After processing, 1 represents the nonedge points, and 0 represents the edge points. Then, the processed edge probability map is sent into the distance field branch, and we will get the predicted distance field map. As for the true distance field map, in the stage of processing training data, we use the distance field function for the ground truth (true edge map) to get it. Finally, MSE is used to calculate the loss value.

### 3.3. Loss Function

For the pixel-level classification problem of edge detection, the classification of each pixel of an image is usually regarded as a binary classification problem (edge points and nonedge points). Therefore, cross entropy is used as the cost function of each pixel classification in RFEN. Each ground truth in the BSDS500 dataset is marked by multiple annotators. As each annotator has a different cognition of the edge, there is some noise in the edge of the dataset. The threshold method is used to exclude the disputed points in the label images. The loss function of each pixel in this model is expressed as

(2) $$\begin{array}{c} f\left(x\right)=\left\{\begin{array}{cc} \alpha \cdot \;\log\left(1-P\left(X_{i};W\right)\right), & \left|lb_{i}\right|=0,\\ 0, & 0<\left|lb_{i}\right|<64,\\ \beta \cdot \;\log\;P\left(X_{i};W\right), & \left|lb_{i}\right|\geq 0, \end{array}\right) \end{array}$$

in which

(3) $$\begin{array}{c} \left\{\begin{array}{c} \alpha =\lambda \cdot \frac{\left|Y^{+}\right|}{\left|Y^{+}\right|+\left|Y^{-}\right|},\\ \beta =\frac{\left|Y^{-}\right|}{\left|Y^{+}\right|+\left|Y^{-}\right|}. \end{array}\right) \end{array}$$

*Y* ^+^ and *Y*^−^ denote the positive sample set and negative sample set, respectively. The hyperparameter *λ* is to balance positive and negative samples. The CNN feature vector and ground truth gray value at pixel *i* are presented by *X*_*i*_ and *lb*_*i*_, respectively. *P*(*X*) is the sigmoid function, and *W* denotes all the parameters that will be learned in RFEN.

In the training process of RCF, the loss of each stage and the loss of the fusion module are taken as the loss function of the whole model. But this simple additive loss function does not reflect the importance of the fusion module. Therefore, we directly use the loss of the fusion module to conduct optimization training on RFEN. The loss function of RFEN is

(4) $$\begin{array}{c} L\left(W\right)=\sum_{i}^{I}l\left(X_{i}^{\text{fuse}};W\right), \end{array}$$

in which *X*_*i*_^fuse^ denotes the activation value of the *i*-th pixel in the image output by the fusion module. |*I*| denotes the total number of pixels in the image.

The distance field branch is ultimately a regression problem. The loss function of the predicted distance field in DF is expressed as

(5) $$\begin{array}{c} L_{1}=\frac{1}{m}\sum_{i=1}^{m}{\left(y_{i}-{\hat{y}}_{i}\right)}^{2}, \end{array}$$

in which

(6) $$\begin{array}{c} {\hat{y}}_{i}=\eta \cdot N\left(G_{i}^{\text{fuse}};W_{1}\right). \end{array}$$

*y*_*i*_ and ${\hat{y}}_{i}$ denote the value of the *i*-th pixel in the true distance field map and predicted distance field map, respectively. *m* is the total number of pixels in the predicted edge probability map. *G*_*i*_^fuse^ denotes the value of *X*_*i*_^fuse^ after threshold processing. *N*(*X*) is the sigmoid function, and *W*_1_ denotes all the parameters that will be learned in DF. The hyperparameter *η* is to set the value of the predicted distance field map between 0 and 5.

The loss function of DF-CNN is expressed as

(7) $$\begin{array}{c} L=L\left(W\right)+\gamma L_{1}, \end{array}$$

in which *L*(*W*) and *L*_1_ denote the loss function of RFEN and DF, respectively. The hyperparameter *γ* is to adjust the proportion of the distance field branch loss function in the overall loss function. In this paper, we set it to 0.5.

## 4. Experiments

We implement our network using the publicly available PyTorch. The ResNet101 model that is pretrained on ImageNet is used to initialize our model. For other SGD hyperparameters, the initial global learning rate is set to 1*e* − 2 and will be adjusted with the number of iterations. The momentum and weight decay are set to 0.9 and 0.0005, respectively. We train the model for 30 epochs. The parameters l*b* and *λ* in loss function are also set depending on training data. The experiments in this paper are implemented using an NVIDIA GTX 1080ti GPU.

The detection indicators of the edge detection model mainly include ODS (optimal dataset scale) and OIS (optimal image scale), in which ODS refers to the detection results when all images in the test set are fixed with the same threshold; OIS refers to the detection result for each image using the optimal threshold for the current image. The edge map output by the model in this paper was processed by non-maximum suppression, and the index was measured by the Edge Box toolkit.

### 4.1. Datasets

In this paper, we use a total of three datasets: BSDS500, PASCAL, and NYUD. However, in order to better illustrate the efficacy of the ablation experiments, we used different combinations of datasets. Next, we describe the three datasets in detail.

BSDS500: the traditional edge detection dataset BSDS500 is composed of three parts: training set, validation set, and test set. Among them, the training set contains 200 pictures, the validation set contains 100 pictures, and the test set contains 200 pictures. To prevent the model from overfitting, the BSDS500 dataset is enhanced. By using the OpenCV toolkit, a total of 300 images from the training set and validation set of BSDS500 were rotated, expanded, and clipped.

PASCAL: as one of the benchmark data, PASCAL is frequently used in edge detection, object detection, image segmentation network comparison experiment, and model effect evaluation. The training set contains a total of 11,530 annotated images, and all of them would be used to train the purposed model.

NYUD: NYUD is an indoor scene dataset; it is made up of 1,449 pairs of aligned RGB and densely labeled depth images. The dataset is split into 381, 414, and 654 images for training, validation, and test, respectively. We train our DF-CNN network with training and validation sets following the settings in RCF. We combine the training and validation set and augment them by rotating the images and corresponding annotations to 4 different angles (0, 90, 180, and 270 degrees) and flip them at each angle.

### 4.2. Ablation Experiments

#### 4.2.1. Different Backbone Networks

**Figure 6:**
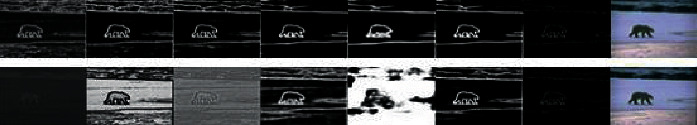
From one to eight columns is the output of stages 1, 2, 3, 4, 5, fuse, NMS, and original images, the top line is the outputs of RCF, and the bottom one is the outputs of our model.

We explore the influence of the backbone of the model by replacing the VGG16 with the ResNet101. Specifically, we use the five stages of the ResNet101 to directly replace the five stages of the VGG network, and we take the fusion layer of all stages of the ResNet101 network as the subsequent input. The results are reported in Table 1. Obviously, under the same training set, after replacing the backbone, our model improves the ODS F-measure from 0.798 to 0.802. We show the intermediate results of each stage from our model and RCF in Figure 6, and we found that the output feature map of each stage of RCF was similar, but the information of the fused feature map was not fully utilized. Although the feature map of the previous stages in our network was not satisfactory, the output effect of the fused edge map was better.

#### 4.2.2. Different Training Sets

As known to all, in deep learning tasks, the training set is the most basic and important factor, which can directly affect the training results. Referred to the previous work, here, we do a comparative experiment; one experiment adopts the BSDS500 dataset as the training set, and the other experiment adopts BSDS500 + PASCAL as the training set. The results are reported in Table 2. In the model of RCF, it improves the ODS F-measure from 0.798 to 0.806, and in our model, it improves the ODS F-measure from 0.802 to 0.813. It is proved that the expansion of training set has a great influence on the improvement of training results.

#### 4.2.3. Increasing the Distance Field Branch

**Figure 7:**
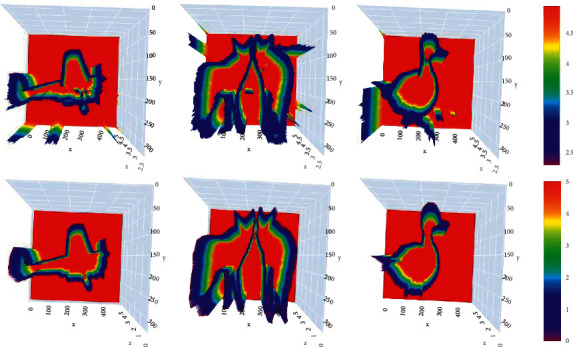
The top line is the outputs of the predicted distance field chromaticity map of the test set, and the bottom one is the true outputs of the distance field chromaticity map of the test set; the color bar represents the chroma corresponding to the distance.

As the concept of distance field branch proposed by us for the first time in this paper, to verify its effectiveness, we add distance field branch to the network on the basis of the above experiment, in which the processing result of RFEN output is taken as the input of the distance field branch. The implementation details are described below. First, we introduced the RFEN parameters in experiment 4.2.2 into the model and froze them. Then, we trained the distance field branch and got a better effect. Finally, the RFEN is defrosted, and the joint training is carried out to get the final result. The comparison results between experiment 4.2.2 that does not have a distance field branch and experiment 4.2.3 (DF-CNN) are shown in Table 3. In this experiment, the model will have two outputs: a predicted edge probability map and the corresponding predicted distance field map. The range of predicted distance field values is 0 to 5; we show the true and predicted distance field chromaticity maps in Figure 7.

### 4.3. Experimental Analysis

**Figure 8:**
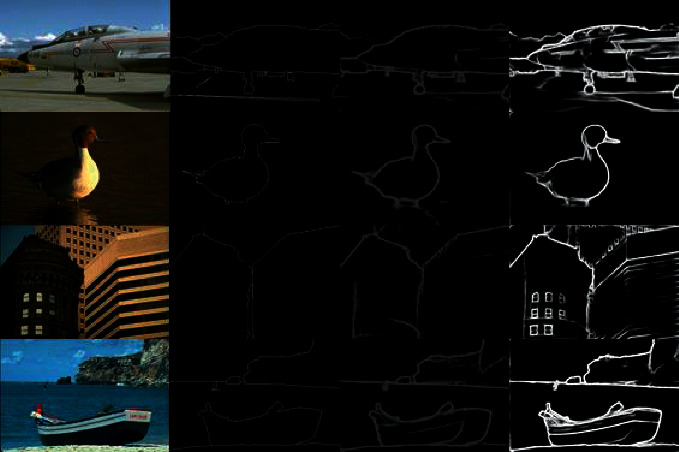
Several examples of the comparison between the edge probability map output by the DF-CNN and the RCF on the BSDS500 tests. From left to right: origin image, ground truth, DF-CNN edge map, and RCF edge map.

We show a statistical comparison in Table 4. From experiment 4.2.2 to experiment 4.2.3, the ODS F-measure increases from 0.813 to 0.818, which proves the validity of the distance field branch. From RCF to DF-CNN, the ODS F-measure increases from 0.806 to 0.818, DF-CNN is 1.2% ODS F-measure higher than RCF. Compared to the latest edge detection technology PiDiNet, as shown in Table 4, the PiDiNet seems to focus more on the speed of edge detection. It clearly demonstrates that our DF-CNN achieves the best performance and becomes the state-of-the-art technology; at the same time, compared with RCF, the speed of our network decreases while the accuracy is improved, but it can also meet the real-time demand. In addition, the comparison between the predicted edge probability map output by the model in this paper, and the original image of BSDS500 is shown in Figure 8.

**Figure 9:**
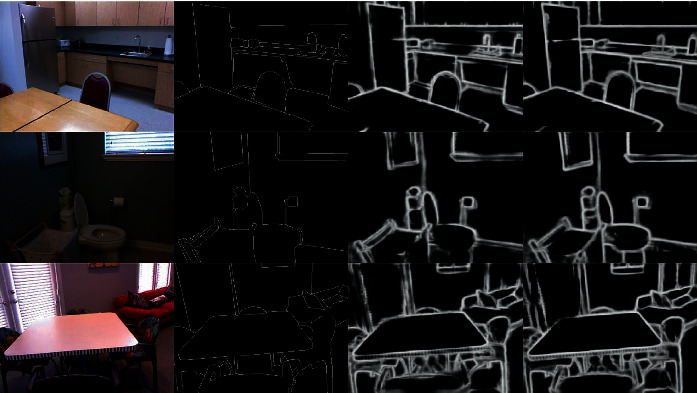
Several examples of the comparison between the edge probability map output by the DF-CNN and the ground truth on the NYUD tests. From left to right: origin image, ground truth, DF-CNN edge map, and RCF edge map.

To prove the effectiveness of DF-CNN, we also train our network on the NYUD dataset. In the training process, *λ* is set to 1.2, and no *η* is needed for NYUD since it only has one ground truth for each image. Other network settings are the same as used for BSDS500. As a testing, we just train the model for RGB images, and some examples of DF-CNN that tested on NYUD are shown in Figure 9.

## 5. Conclusions

This paper presents a new edge detection model named DF-CNN. Based on the ideas of RCF and HED, the model replaces the VGG16 with ResNet101 to improve the expression ability of the backbone and fully integrate multiscale features. Finally, the performance of the model is improved by combining the feature map generated by the fusion module that makes full of semantic and fine detail features with the distance field branch. Experiments show that this model can effectively generate high-quality edge images and the corresponding distance field map; although the speed is somewhat lower than the RCF, the real-time performance of the model is guaranteed.

Further consideration is given to the result of distance field branch output by the model. Therefore, we consider that it can be applied to the instance segmentation task. Since the result of the current instance segmentation method often presents the problem of rough boundary segmentation, the fusion of the distance field branch into the segmentation task can achieve the purpose of improving the segmentation accuracy. Subsequent work will be carried out along with Mask R-CNN, and the method of detection before segmentation will be adopted. The research scheme will be detailed below.

Adhering to the principle of the first detection and then segmentation, we can get a predicted mask from the model. The predicted mask is taken as input to the distance field branch. Finally, we will obtain two losses: mask loss and DF loss; then, the sum of the two is taken as the total loss of the distance field mask branch. The specific realization idea of distance field branch in the instance segmentation model is different with DF-CNN because the edge curve in the instance segmentation is closed and continuous; when we make the true distance field label, referencing the traditional active contour model, we set the edge point value to 0; a point beyond the edge is given a positive value based on its distance from the nearest edge point; similarly, points within the edge are given a negative value based on their distance from the nearest edge point. A convolutional neural network is used to learn the distance field branch and fit the distance field function; it is expected that the high-quality distance field map can be used to obtain a more accurate segmentation edge.

## Figures and Tables

**Table 1:** The comparison results with different backbone networks.

Experiment	Backbone network		Train dataset		Evaluation results	
	VGG16	ResNet101	BSDS500	PASCAL	ODS	OIS
Ref [19]	√		√		0.798	0.815
Experiment 4.2.1		√	√		0.802	0.818

**Table 2:** The comparison with some competitors on different datasets.

Experiment	Backbone network		Train dataset		Evaluation results	
	VGG16	ResNet101	BSDS500	PASCAL	ODS	OIS
Ref [19]	√		√		0.798	0.815
Ref [19]	√		√	√	0.806	0.823
Experiment 4.2.1		√	√		0.802	0.818
Experiment 4.2.2		√	√	√	0.813	0.831

**Table 3:** The comparison results between experiment 4.2.2 that does not have a distance field branch and experiment 4.2.3 (DF-CNN).

Experiment	Backbone network		Distance field branch	Train dataset		Evaluation results	
	VGG16	ResNet101	DF	BSDS500	PASCAL	ODS	OIS
Experiment 4.2.2		√		√	√	0.813	0.831
Experiment 4.2.3 (DF-CNN)		√	√	√	√	0.818	0.833

**Table 4:** The statistic comparison with some competitors on BSDS500 dataset.

Method	ODS	OIS	FPS
Canny [7]	0.611	0.676	28
EGB [23]	0.614	0.658	10
MShift [24]	0.598	0.645	1/5
gPb-UCM [25]	0.729	0.755	1/240
Sketch Tokens [26]	0.727	0.746	1
MCG [4]	0.744	0.777	1/18
SE [9]	0.743	0.763	2.5
OEF [27]	0.746	0.770	2/3
DeepContour [16]	0.757	0.776	1/30†
DeepEdge [17]	0.753	0.772	1/1000†
HFL [28]	0.767	0.788	5/6†
N4-Fields [29]	0.753	0.769	1/6†
HED [18]	0.788	0.808	30†
RDS [30]	0.792	0.810	30†
CEDN [31]	0.788	0.804	10†
MIL + G-DSN + MS + NCuts [32]	0.813	0.831	1
RCF [19]	0.806	0.823	30†
RCF-MS [19]	0.811	0.830	8†
Experiment 4.2.1	0.802	0.818	17†
Experiment 4.2.2	0.813	0.831	17†
Experiment 4.2.3 (DF-CNN)	0.818	0.833	16†
PiDiNet [20]	0.807	0.823	92†
PiDiNet-L [20]	0.800	0.815	128†
PiDiNet-Small [20]	0.798	0.814	148†
PiDiNet-Tiny [20]	0.789	0.806	152†

† means GPU time.

## Data Availability

The datasets and results generated during the study are available at https://github.com/Hudadan666/DF-CNN.
